# Evidence for competition and cannibalism in wormlions

**DOI:** 10.1038/s41598-021-92154-7

**Published:** 2021-06-17

**Authors:** Inon Scharf, May Hershkovitz Reshef, Bar Avidov, Ofer Ovadia

**Affiliations:** 1 School of Zoology, Faculty of Life Sciences, Tel Aviv University, 69978 Tel Aviv, Israel; 2 Department of Life Sciences, Ben-Gurion University of the Negev, Beer-Sheva, Israel

**Keywords:** Ecology, Behavioural ecology, Animal behaviour

## Abstract

Trap-building predators, such as web-building spiders and pit-building antlions, construct traps to capture their prey. These predators compete over sites that either enable the construction of suitable traps, are prey rich, or simply satisfy their abiotic requirements. We examined the effect of intraspecific competition over suitable space in pit-building wormlions. As expected, the ability of wormlions to select their favorable microhabitats—shaded or deep sand over lit or shallow sand—decreased with increasing density. Favorable microhabitats were populated more frequently by large than by small individuals and the density of individuals in the favorable microhabitat decreased with their increase in body mass. The advantage of large individuals in populating favorable microhabitats is nevertheless not absolute: both size categories constructed smaller pits when competing over a limited space compared to those constructed in isolation. The outcome of competition also depends on the type of habitat: deep sand is more important for large wormlions than small ones, while shade is similarly important for both size classes. Finally, in contrast to previous reports, cannibalism is shown here to be possible in wormlions. Its prevalence however is much lower compared to that documented in other trap-building predators. Our findings show that the advantage of large individuals over small ones should not be taken for granted, as it can depend on the environmental context. We present suggestions for the relative lack of competitive advantage of large wormlion individuals compared to other trap-building predators, which may stem from the absence of obvious weaponry, such as sharp mandibles.

## Introduction

Animals and plants compete directly or indirectly over shared, limited resources, and such competitive interactions are prevalent in nature^1,2^. Competition is common when the overlap in niche space is high. For example, preference for the same food type, activity time, or habitat can lead to strong competition, which can be relaxed by food, spatial, or temporal partitioning, consequently allowing coexistence^3,4^. Individuals of the same species share more axes of their niche than heterospecifics and, thus, intraspecific competition is usually stronger than interspecific competition^5,6^. When given a choice between habitats of varying quality, animals first select the high-quality habitat, and the lower quality ones are utilized only with increasing population density; hence, habitat quality is an outcome of both its resource abundance and the number of resident competitors and predators^7–9^. Basic habitat selection models assume that individual variation in competitive ability can be ignored or abstracted. This assumption, however, was relaxed in more realistic models that incorporate the superior competitive advantage of specific individuals or different strategies, while examining the consequences on the population densities of the focal species in habitats of varying qualities (reviewed in^10^).

Large individuals of the same species are often competitively superior to small ones^11^. This is expressed, for example, in more intense reproduction for large females, more mating opportunities for large males, and dominance over food resources for both sexes^12–14^. The selection pressures that maintain small adult size are either the longer time and/or the more intense foraging required to grow large, which impair survival^15–17^. Considering competing juveniles, large individuals are superior to small ones, dominating food resources and suitable habitats^18,19^. Furthermore, in many arthropods and amphibians, competition is frequently manifested in cannibalism, and it is usually the large individuals that prey on the smaller ones, adding an important benefit to being large^20–22^. Competition for suitable habitats also depends on the order of arrival and individuals settling earlier may have a “priority effect”, even if they are otherwise less competitive in direct interactions^23,24^. The magnitude of such priority effects depends on several factors, such as the interval length between arrivals, or the presence of predators^25,26^.

Sit-and-wait predators save the energetic cost of movement by choosing an ambush site, where they wait for the prey to reach them^27^. Hence, there is great importance in choosing a suitable ambush site, characterized by favorable abiotic conditions (e.g., temperature or proximity to the water), or one that facilitates the capture of prey^28–30^. Trap-building (hereafter, TB) predators belong to a sub-group of sit-and-wait predators that construct a trap to catch their prey^31,32^. In addition to considerations related to abiotic conditions and prey abundance, TB predators should choose sites that require the least investment of energy in the construction and maintenance of their traps^33,34^. Relocation after initial trap construction is undesirable owing to the cost of constructing a new trap^35,36^. Furthermore, trap relocation is often dangerous, making the relocating predator vulnerable to other predators and harmful abiotic conditions^37,38^. Consequently, there is competition among TB predators over the most suitable sites for trap construction, and in such competition, large individuals are superior to small ones^39–41^. In addition to this direct competition over ambush sites, a TB predator can block the way of potential prey from reaching the traps of other predators. This process, termed “shadow competition”, assumes that sites in the periphery of the TB predators' cluster receive more prey than sites in the center^42–44^. Finally, many TB predators, especially spiders and antlions, prey on whatever prey is caught in their trap, including related species and conspecifics^45–48^. The outcome of such cannibalistic and intra-guild predation attempts strongly depends on body size (larger individuals prey on smaller ones^22,49,50^).

**Figure 1:**
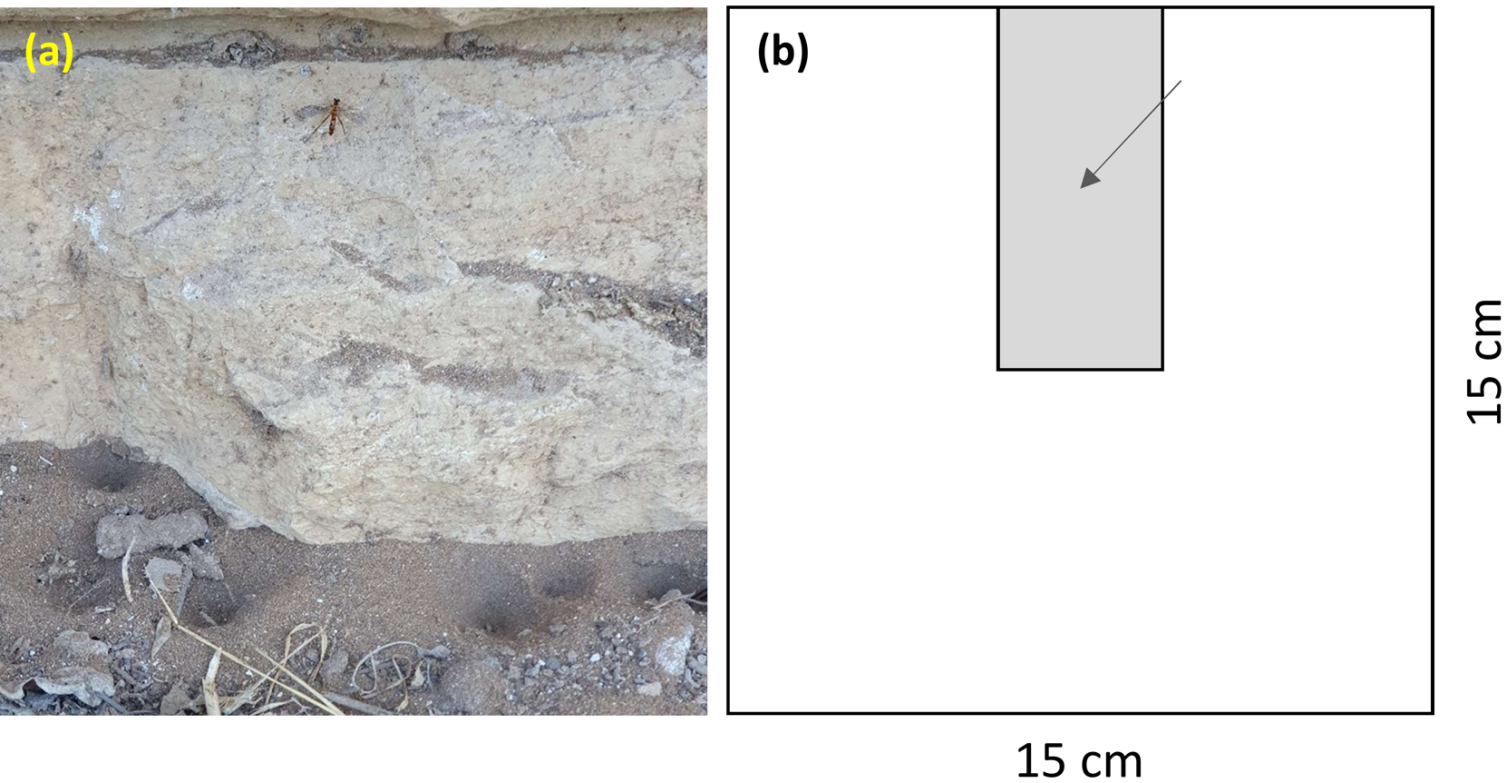
(**a**) Several pits constructed by wormlions beneath buildings at Tel Aviv University, Tel Aviv, Israel. An adult wormlion female is present on the wall, perhaps before or after oviposition (photo taken by the first author). (**b**) A scheme of the tray used for Experiments 1–4. Wormlions were always placed in the center of the rectangular area (marked with an arrow). The rectangular area (gray) in most cases contained better conditions than its surroundings (white), either shaded vs. lit sand or deep vs. shallow sand.

Wormlions are fly larvae that dig pit-traps and ambush their prey^51^ (Fig. 1a). Owing to their pit-trap construction, their foraging strategy is similar to that of pit-building antlions, which present together an example of convergent evolution^52,53^, although several differences exist between the taxa. For example, antlions use spiral digging, while wormlions use central digging, which is less efficient, and antlions can catch larger prey than can wormlions of the same size^54,55^. Furthermore, while antlions are known to be cannibalistic^22,45,46^, there is only a single anecdotal report to date, suggesting that cannibalism does not exist in wormlions^56^. Previous studies have demonstrated wormlion preference for fine-textured loose soil, deep and dry sand, and shade^57–59^. The shade is preferred in order to avoid exposure to high temperature and desiccation^60^, while deep sand enables the construction of larger pits, which in turn improve prey capture^61^. Similar to other TB predators, wormlions compete over suitable sites in which to construct their traps, and the outcome of the competition for the loser is either to settle in an inferior microhabitat or to construct a smaller pit-trap^52,58^.

We examined here competition over favorable microhabitats between wormlions of equal and of different sizes. We predicted that the proportion of wormlions settling in inferior microhabitats should increase with their density. We also predicted that the competitively superior large wormlions would occupy the more favorable microhabitats, while the competitively inferior small individuals would construct their traps in the less preferred microhabitats. If a priority effect exists, this pattern is expected to be weaker when the smaller individuals arrive at the favorable microhabitat before the larger ones. Finally, we predicted that smaller wormlions would be more affected by competition under limited space than larger ones, i.e., they would reduce their pit size to a greater extent, as competition under such conditions becomes asymmetrical according to the different sizes of the competitors.

## Materials and methods

Wormlions (*Vermileo* sp.) were collected from loose soil patches located next to buildings providing cover, at Tel Aviv University, Tel Aviv, Israel (Fig. 1a). We focused on shade and deep sand in the following experiments, both of which are preferred by wormlions in choice settings^57,58^. The experimental arena in Experiments 1–4 was an aluminum tray, 15 × 15 cm, filled with either 2 cm sand (deep sand) or 0.5 cm sand (shallow sand). The wormlions were placed in the middle of a rectangular area (7.5 × 3.5 cm) adjacent to one of the tray edges (Fig. 1b). The abiotic conditions in this rectangle differed from those of the surroundings, as explained below. The experimental cup in Experiments 5–6 comprised a cup with a diameter of 5.5 cm filled with 2 cm of sand. The sand used in all experiments was of particle size < 250 µm, reflecting the wormlion preference for fine-textured sand. The goal of experiments 1 and 3 was to examine the effect of density on the ability of wormlions to settle in their preferred microhabitats (shaded over lit and deep over shallow microhabitats). We tested also the effect of density in homogenous microhabitats (shaded, lit, deep, or shallow; treatments 2 and 3 in each experiment) as a reference, to examine the tendency of wormlions to remain in their initial placement location when only density is changing.

### Experiments 1–2: competition over shade

#### Experiment 1: competition among wormlions of a similar size

We collected 265 wormlions and weighed them (accuracy of 0.1 mg; 8.9 ± 5.4 mg, mean body mass ± 1 SD). We first sorted the collected individuals according to body mass and then divided them into groups of 1, 2, 3, 4, or 5 individuals, so that the within-group variances in body mass were minimal. We randomly allocated individuals to one of the following three treatments: (1) the rectangular area was shaded, while the rest of the tray was lit (hereafter, the shade choice treatment); (2) the entire tray was shaded; and (3) the entire tray was lit. Each of the 15 treatment combinations (treatment × density) was replicated 4–8 times (5.7 ± 1.1, mean number of trays ± 1 SD). In all cases, the sand depth was 2 cm. We photographed the tray after 24 h, documented the number and location of the constructed pits, and then measured their area using ImageJ^62^.

#### Experiment 2: competition between small and large wormlions

We collected 288 wormlions and weighed them (7.2 ± 5.8 mg, mean body mass ± 1 SD). We first sorted the collected individuals according to body mass and assigned individuals to three different treatments, such that the body mass differences among treatments were minimized. Then, we sorted again the individuals according to treatment and body mass. We cut the mass-sorted list in the middle, resulting in two groups, small individuals (first half) and large individuals (second half), per treatment. We matched wormlions in groups of four—two small and two large individuals, in ascending order (e.g., the two smallest individuals of the half dataset of smaller individuals were matched with the two smallest individuals of the half dataset of the larger individuals). The difference in body mass between the two large and the two small larvae was 9.0 ± 3.8 mg (mean ± 1 SD), meaning that they most likely pertained to different instar stages. The area below a rectangular cover was shade, while the rest of the tray was lit (similar to treatment 1 in Experiment 1). The three treatments were: (1) all four wormlions were placed simultaneously under the shaded area, (2) the two small ones were placed 2 h before the large ones under the shaded area, and (3) the two large ones were placed 2 h before the small ones under the shaded area. Each of the three treatments was replicated 24 times (3 treatments × 24 replications = 72 experimental trays). After 24 h, we documented the wormlions’ location (shade/light), their identity (small/large), and measured their pit area. Not all wormlions constructed pits, and larger individuals constructed pits more frequently than smaller individuals. Therefore, we referred only to the identity of the individuals constructing pits and calculated for each tray the expected number of large individuals under shade assuming no difference according to size (‘null expectation’). We compared this expected number to the observed number of large individuals under shade. For example, let us assume that three individuals constructed pits, of which two were large individuals and the third was a small individual. Of these three pits, two were constructed in the shaded area, by large individuals. Therefore, the ‘null expectation’, assuming no difference according to size, is that 2/3 of the pits under shaded area would be constructed by large wormlions. However, the observed proportion of large wormlions constructing their pits in the shaded area is one. See the Supplementary Material for a description of all possible cases.

### Experiments 3–4: competition over deep sand

#### Experiment 3: competition among wormlions of a similar size

We collected 252 wormlions and weighed them (6.5 ± 4.5 mg, mean body mass ± 1 SD). We first sorted the collected individuals according to body mass and then divided them into groups of 1, 2, 3, 4, or 5 individuals, so that the within-group variances in body mass were minimal. We randomly allocated individuals to one of the following three treatments: (1) the rectangular area contained deep sand (2 cm), while the rest of the tray contained shallow sand (0.5 cm; hereafter, the depth choice treatment); (2) the whole tray contained deep sand; and (3) the whole tray contained shallow sand. Each of the 15 treatment combinations (treatment × density) was replicated 4–6 times (5.5 ± 0.6, mean number of trays ± 1 SD). The trays were placed under shade, 12:12 L:D. After 24 h, we documented the wormlions’ location (deep/shallow sand) and measured their pit area.

#### Experiment 4: competition between small and large wormlions

We collected 288 wormlions and weighed them (8.5 ± 6.5 mg, mean body mass ± 1 SD). We sorted them into sizes and assigned them to treatments as in Experiment 2. The difference in body mass between the two large and the two small larvae was 10.3 ± 3.5 mg (mean ± 1 SD). The rectangular area contained deep sand, while the rest of the tray contained shallow sand (similar to treatment 1 in Experiment 3). We randomly assigned the groups to one of the following two treatments: (1) all four wormlions were placed simultaneously on the deep sand, (2) the two small ones were placed 2 h earlier than the large ones, all on deep sand, and (3) the two large ones were placed 2 h earlier than the small ones, all on deep sand. Each of the three treatments was replicated 24 times (3 treatments × 24 replications = 72 experimental trays). After 24 h we documented the wormlions’ location, their identity (small/large), and measured their pit area. We calculated the proportion of large wormlions constructing their pits in the deep sand out of the total number of individuals that constructed pits in the deep sand and compared this proportion to the expected probability according to the total number of pits constructed (see Experiment 2).

### Experiment 5: competition between small and large wormlions over a limited area

We collected 100 wormlions and weighed them (10.1 ± 7.5 mg, mean body mass ± 1 SD). We then sorted the collected individuals according to body mass and allocated pairs of one small and one large individual to an experimental cup (N = 50 pairs). The difference in body mass between the large and small wormlion in each cup was 12.6 ± 3.3 mg (mean ± 1 SD). Fifty percent of the pairs were placed as one pair to each cup, while the other pairs were separated and placed in two individual cups. After 24 h, we measured the area of the pits constructed. The next day, we switched between the treatments: the pairs that had shared a cup were separated, and the pairs that had been kept separated were placed together in the same cup. The pit area was measured, as before, after 24 h. The procedure yielded two measurements per individual: the pit area when constructed alone and when paired with a different sized competitor (a small competitor if it was a large wormlion and a large competitor if it was a small wormlion).

### Experiment 6: cannibalism

In Experiment 5, we observed two cases of cannibalism, in which the large wormlion preyed on the smaller one (a mass difference between the cannibal and the victim of 7.5 mg and 11.0 mg). Because previous studies on wormlions had doubted the existence of cannibalism, we extended the number of pairs placed together in the same experimental cup. In total, we have data for 139 pairs. The body mass of small larvae ranged from 0.1 to 8.9 mg (2.9 ± 2.2 mg, mean body mass ± 1 SD) and that of large larvae from 4.1 to 31.9 mg (13.8 ± 5.7 mg, mean body mass ± 1 SD). The body mass difference between paired wormlions ranged from 3.7 to 23.2 mg (10.9 ± 3.8 mg; mean ± 1 SD). After 24 h, we documented the prevalence of cannibalism by observing any corpses under a stereomicroscope and weighing the remaining larva to verify that it had gained mass.

### Statistical analysis

To analyze the number of pits constructed in Experiments 1 and 3, we employed a hierarchical generalized linear model, using Poisson distribution and log link function. The number of pits constructed was treated as the response variable, and the number of wormlions in the experimental tray, mean body mass per tray, and treatment were included in the statistical model as explanatory variables. The categorical variable treatment was converted into a dummy variable. Since it comprised three levels, its inclusion in the statistical model required generating two binary indicator variables, representing two of the three treatment levels, with each one of them being compared with the reference level, i.e., the third treatment group. The pit area in Experiments 1–5 was analyzed employing a hierarchical generalized linear model, using Normal distribution and an identity link function. The test is "hierarchical" because the tray/cup was included in the statistical model as a random factor (i.e., accounting for the dependency of individuals within tray/cup). Pit area was treated as the response variable. In Experiments 1 and 3, wormlion density, mean body mass, treatment, and location were treated as the explanatory variables. In Experiments 2 and 4, treatment (individuals placed simultaneously or not), location, and size (large/small) were treated as explanatory variables. In these two experiments, we also used repeated-measures ANOVAs to compare the observed and expected proportion (arcsin-transformed) of large wormlions constructing their pits in the initial location (as the response variable), with treatment as the between-subject factor. In Experiment 5, size (large/small), treatment (alone/together), and order of treatments (first alone or together) were treated as the explanatory variables. Experiment 6 was analyzed using two logistic regressions, with the occurrence of cannibalism as a binary response variable, the body mass difference between the paired wormlions as the explanatory variable, and either the mass of the small individual or the mass of the large individual as another explanatory variable. When an interaction was not significant, it was removed, and the test was redone. All analyses were conducted using STATA 15 (2017; StataCorp, College Station, TX).

## Results

### Experiments 1–2: competition over shade

#### Experiment 1: competition among wormlions of a similar size

**Figure 2:**
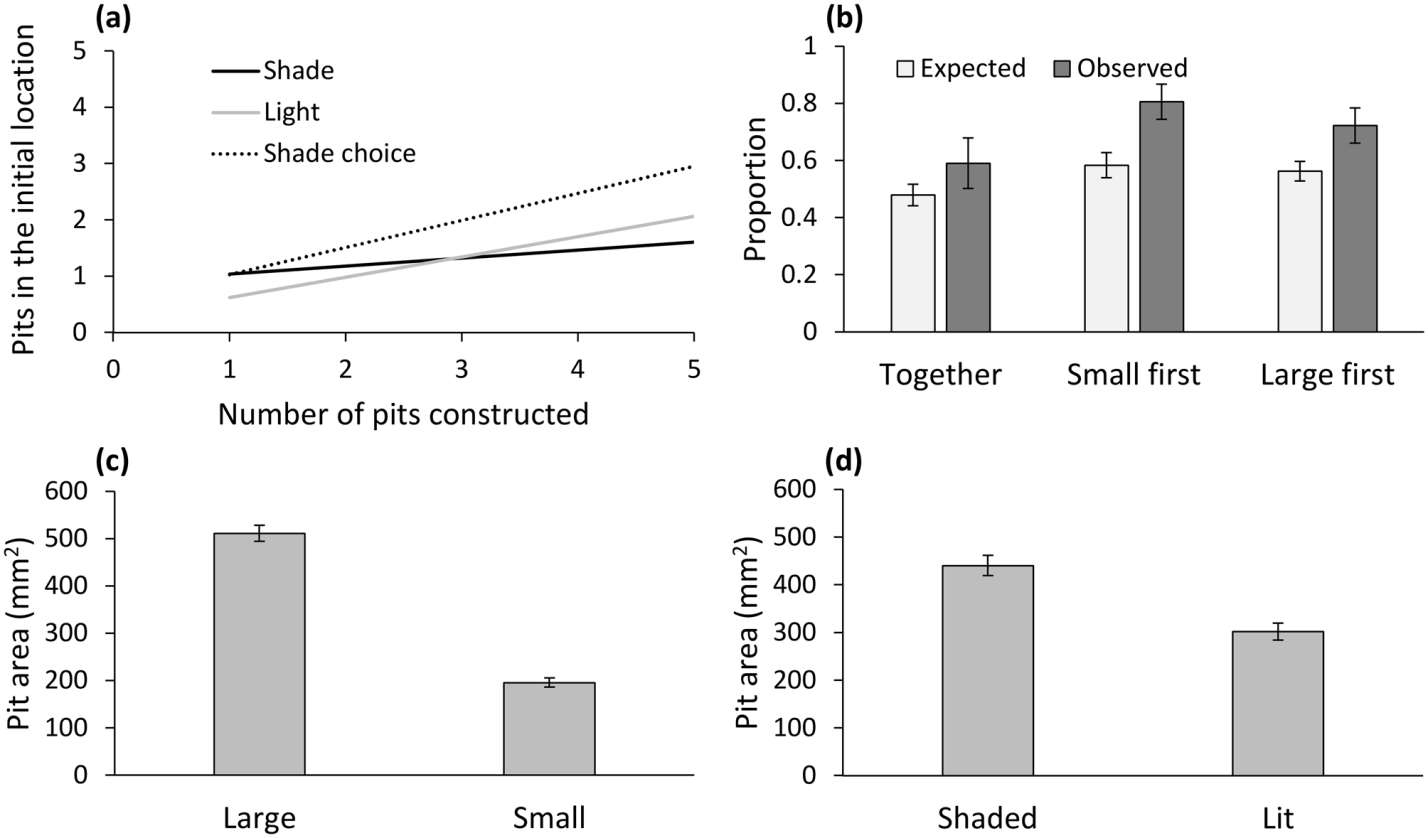
(**a**) The interactive effect of treatment (shade, light, or shade choice treatments) and the total number of pits in the tray on the number of pits constructed in the initial placement location. Only regression lines are shown for simplicity. (**b**) Large wormlions remained in the initial placement location (under shade) more frequently than expected by chance, independent of whether all wormlions were placed simultaneously or not. (**c**) Pits constructed by larger wormlions were larger than those constructed by smaller ones. (**d**) Pits constructed in shaded sand were larger than those constructed in lit sand. Means ± 1 SE are presented.

The increase in the number of pits constructed in the initial placement location, as a function of the total number of pits in the tray, was stronger in the shade choice treatment (z = 2.37, *P* = 0.018), and in the lit tray treatment (z = 2.11, *P* = 0.035), compared to that of the shaded tray treatment (Fig. 2a). Increased body mass led to a decrease in the number of pits constructed in the initial placement location (z = − 2.43, p = 0.015). Pit area increased with body mass (z = 5.70, *P* < 0.001), but did not differ among the three treatments (*P* > 0.080). The interaction between pit location and treatment was not significant (*P* > 0.070 in both comparisons).

#### Experiment 2: competition between small and large wormlions

Large wormlions constructed pits in the preferred shaded area more frequently than small wormlions (F_1,69_ = 24.88, *P* < 0.001; Fig. 2b). There was no effect of treatment (placing the small wormlions 2 h earlier or later vs. all simultaneously) on the outcome (F_2,69_ = 0.485, *P* = 0.618). Regarding pit area, large wormlions constructed larger pits (z = 16.25, *P* < 0.001; Fig. 2c). Additionally, pit area was larger in the shaded area of the tray (z = 4.02, *P* < 0.001; Fig. 2d). There was no difference in pit area when small wormlions were placed earlier versus all placed simultaneously and when large wormlions were placed earlier vs. all simultaneously (z = − 0.83 and 0.48, *P* = 0.407 and *P* = 0.628, respectively). All two-way interactions were not significant and hence removed (*P* > 0.062).

### Experiments 3–4: competition over deep sand

#### Experiment 3: competition among wormlions of a similar size

**Figure 3:**
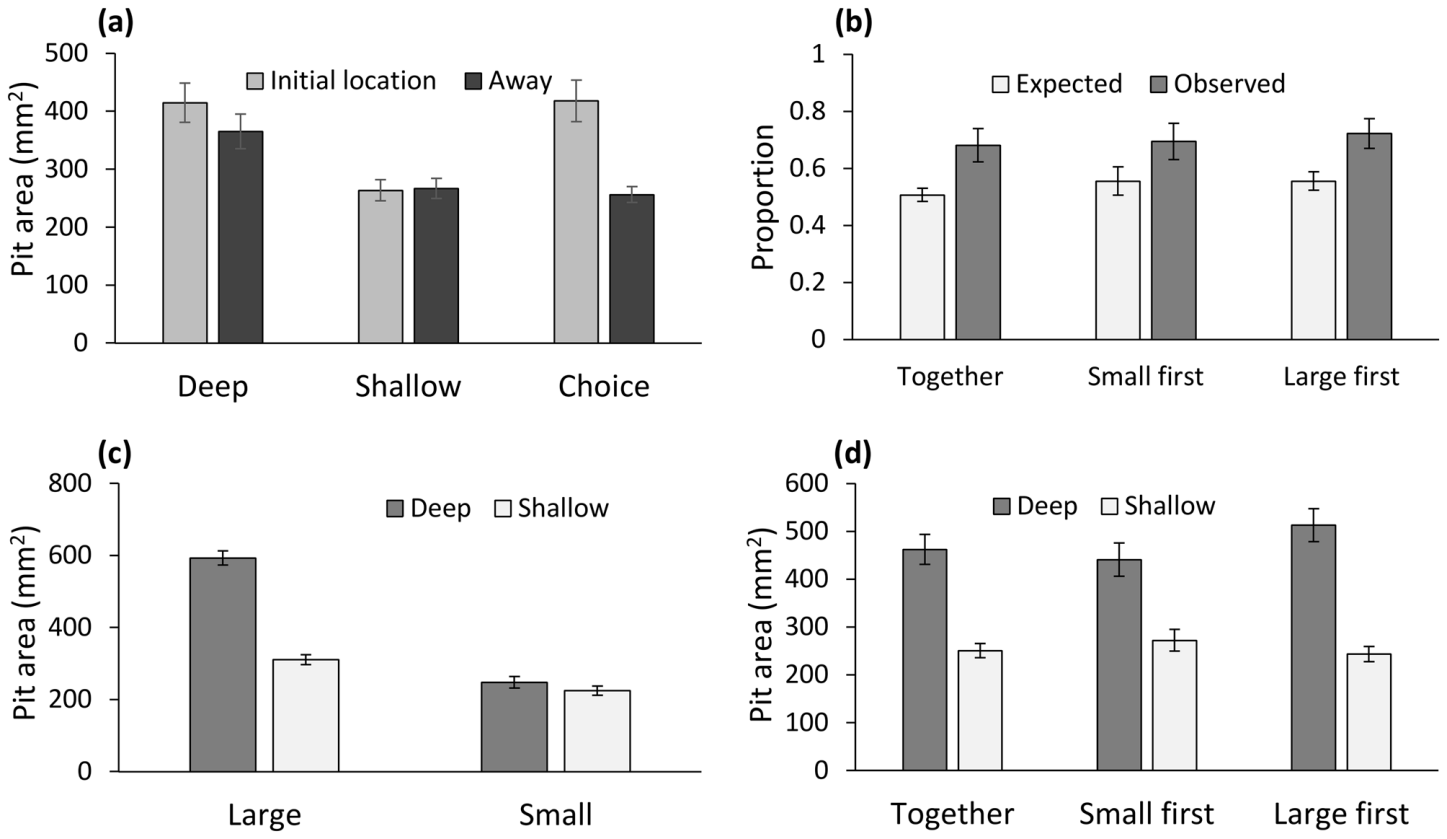
(**a**) Pit area was dictated by sand depth: larger and smaller in the deep and shallow sand treatments, respectively, and variable in the choice treatment, depending on whether deep or shallow sand was chosen. (**b**) Large wormlions remained in the initial placement location (in deep sand) more frequently than expected by chance, independent of whether all wormlions were placed simultaneously or not. (**c**) Pits constructed by large and small wormlions in shallow sand were of a similar area, but large wormlions constructed larger pits in deep sand. (**d**) The interaction between treatment (whether wormlions were placed simultaneously or not) and the difference between pit area in deep and shallow sand. The largest difference was obtained when the large wormlions were placed 2 h before the small ones. Means ± 1 SE are presented.

As expected, the number of pits constructed in the initial placement location was positively correlated with the total number of pits in the tray (z = 2.23, *P* = 0.026). However, there were no differences in the number of pits constructed in the initial placement location between the deep sand and the two other treatments (*P* > 0.835 in both cases), and none of the two-way interaction terms were significant (*P* > 0.660 in both cases). Increased body mass led to a decrease in the number of pits constructed in the initial placement location (z = − 2.40, *P* = 0.016). Pits constructed in the initial placement location were larger than those constructed elsewhere in the tray (z = 3.43, *P* = 0.001), and this pattern was more pronounced in the depth choice treatment (z = 2.28, *P* = 0.022; Fig. 3a). Pits constructed in the deep sand treatment were larger than those constructed in the depth choice treatment (z = − 2.33, *P* = 0.020), and also tended to be larger than those constructed in the shallow sand treatment (z = − 1.89, *P* = 0.058). The effect of pit location on pit area did not differ between the deep and shallow sand treatments (z = − 1.83, *P* = 0.067). As expected, pit area increased with body mass (z = 9.02, *P* < 0.001).

#### Experiment 4: competition between small and large wormlions

Large wormlions constructed pits in the preferred deep sand more frequently than small wormlions (F_1,69_ = 34.76, *P* < 0.001; Fig. 3b). There was no significant effect of treatment (placing the small wormlions 2 h earlier or later vs. all simultaneously) on the outcome (F_1,69_ = 0.12, *P* = 0.889). Regarding pit area, large wormlions constructed larger pits, with this pattern being more pronounced in deep sand (body size × sand depth interaction; z = 6.68, *P* < 0.001; Fig. 3c). Size as main effect was significant as well (z = 3.22, *P* = 0.001), while sand depth was not (z = 0.43, *P* = 0.668). Treatment interacted with sand depth to affect pit area (z = 2.03, *P* = 0.042): Pits in deep sand were larger, when large wormlions arrived 2 h before the small wormlions, compared to a simultaneous arrival (Fig. 3d). All other two-way interactions were not significant and hence removed (*P* > 0.131).

### Experiment 5: competition among small and large wormlions over a limited area

**Figure 4:**
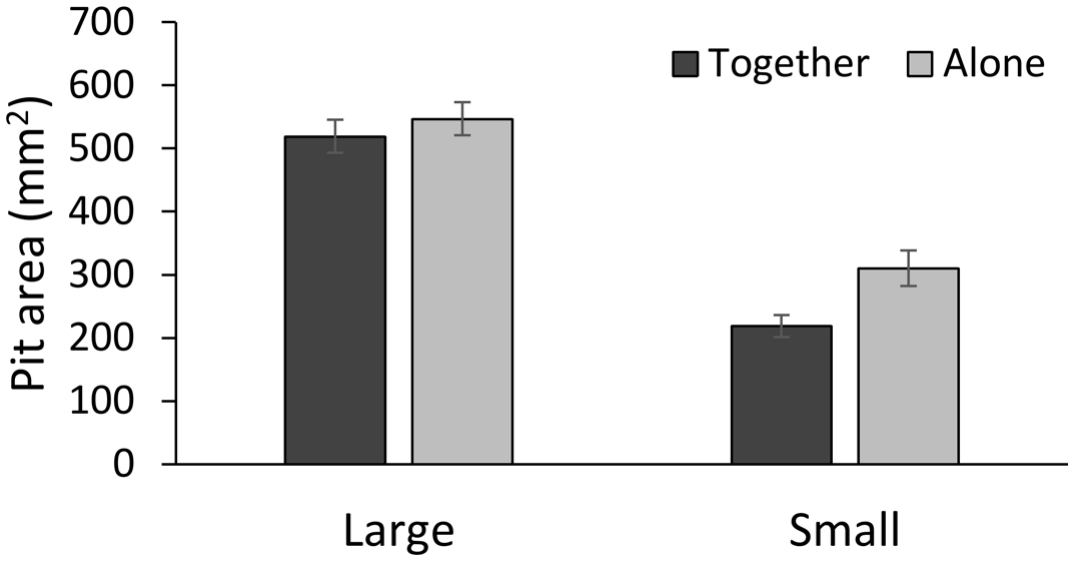
The area of constructed pits was smaller when wormlions were placed together under limited space than when placed alone. This decrease in the area was uniform among both large and small individuals. Means ± 1 SE are presented.

Larger individuals constructed larger pits (z = 7.38, *P* < 0.001). Pit area of both was smaller when the two wormlions were together than when alone (z = − 2.87, *P* = 0.004; Fig. 4), and this pattern held true for both large and small wormlions (z = 1.24, *P* = 0.214). Treatment order, first together or first alone, had no significant effect on pit area (z = − 1.67, *P* = 0.095).

### Experiment 6: cannibalism

Cannibalism took place in 6 out of the 139 pairs, the larger wormlion always being the cannibal (see photos in the Supplementary Material). Neither the body mass of the small wormlion, nor the body mass of the large wormlion, nor the difference in their body masses affected the probability of cannibalism (z = 1.77, *P* = 0.077; z = 1.66, *P* = 0.097; z = 1.41, *P* = 0.157). However, the absence of significant results probably stems from the limited number of positive cases. All the victims were smaller than 3.0 mg, with 40% of the small larvae being larger than this threshold.

## Discussion

Our study provides multiple evidence for competition over space in wormlions, especially over favorable sites. As expected, large individuals were superior to small ones—they occupied the favorable sites, forcing the small individuals to relocate further away, with no "priority effect" evident. In other words, allowing small individuals to arrive earlier did not moderate the advantage of large wormlions in populating superior sites. The advantage of large individuals however was not absolute: they did not prevent neighboring small wormlions from constructing pits, and consequently had to reduce their own pit dimensions. Wormlion cannibalism occurred, although it was rare and required a large body mass difference between the cannibal and its victim. Overall, although large wormlions demonstrated superiority over small ones, we conclude that large individuals have only a moderate negative influence on small ones regarding habitat selection, compared to other TB predators, such as antlions and spiders.

At low densities, wormlions first inhabit the favorable sites, while sites of a lower quality are occupied only when density increases. This process of habitat selection is common in many other animals^9,63,64^. The superiority of shaded over lit microhabitats from the wormlions' perspective is demonstrated in the greater number of pits built in the initial placement location under shade, when the surrounding microhabitat was lit, over a completely shaded microhabitat. In other words, elevating the quality of the favorable microhabitat (shaded and close by) over the inferior microhabitat (lit and more distant) leads to higher densities occupying the former, as demonstrated in pairwise comparisons of three or more habitats of different quality^65,66^. While this pattern is known, the effect of body size/mass has been less often studied. Here, we demonstrate that fewer wormlions remained in the favorable microhabitat as body size increased, as evidence of intensifying competition for space among larger individuals. This finding highlights the importance of referring to body size/mass in the process of habitat selection and might explain the differences obtained among studies examining individuals of the same species but differing in their sizes.

We present evidence of the superior competitive ability of large individuals over smaller ones and demonstrate its consequence for habitat selection. Larger individuals showed a higher probability to occupy the favorable microhabitats, similar to findings in other studies^40,67–69^, and supporting unequal competitor models of habitat selection (reviewed in^10^). Because density in the favorable microhabitat decreases with body mass, and large individuals populate this microhabitat more often, favorable microhabitats might seem less populated than expected, or the inferior microhabitats more populated than expected. This pattern is common too in other animals, with several explanations having been suggested, such as interference or perceptual limitations^70,71^. In some systems of TB predators, large individuals are located in the cluster’s center, and smaller individuals move to the periphery^72–74^. It has been suggested that since prey arrives from the periphery, the exterior positions receive more prey, which is prevented from accessing the cluster’s center, a process called “shadow competition”^42–44^. Large individuals may nonetheless remain in the center more often than small ones because they are less strongly affected by shadow competition than are small individuals^40^. However, here we present a different mechanism behind the occupation of the center by large individuals: if the habitat is not homogenous, and its center is of a higher quality than the periphery, large individuals will aggregate there. The assumption that the center often provides better conditions than the periphery is supported in other systems too because the periphery is more susceptible to various biotic and abiotic types of interference^75,76^.

We expected that the earlier arrival of smaller individuals to the favorable area would moderate the advantage larger individuals have in occupying such superior positions. We also expected that the earlier arrival of larger individuals would strengthen the advantage of larger individuals over smaller ones. Neither expectations held true, and large individuals occupied the superior positions in similar proportions, independent of the order of arrival. This finding is not in accord with the phenomenon of "priority effect", according to which early arrival allows specific animals to occupy the best sites, while late arrivals compromise on inferior sites, with consequences for reproduction and survival^25,77^. However, when large individuals were placed before the smaller ones, the pits constructed in deep sand were moderately larger. Since most pits in deep sand were constructed by large wormlions, this result may also be interpreted as weak, partial support for the priority effect from the perspective of large wormlions: when large ones are introduced first, they can construct larger pits in deep sand, compared to the two other scenarios (either a simultaneous arrival or arrival after the small wormlions). When competing over a limited area, both large and small individuals constructed smaller pits compared to their pits when dug in isolation. The competitive ability of large wormlions is thus limited. This is especially true compared to other TB predators. For example, when forced together in a limited area, large and small individuals of the antlion *Macroleon quinquemaculatus* constructed larger and smaller pits than expected, respectively^39^. Small colonial spiders of the species *Metepeira incrassata* postpone the construction of their webs and allow large individuals to construct their webs first, to prevent potential conflict^73^.

We present here the first evidence of cannibalism in wormlions, in contrast to a previous suggestion that wormlions are not cannibalistic^56^. Cannibalism rates were nevertheless low: ~ 4% of pairs of heterogeneous sizes. In comparison, other TB predators demonstrate much higher cannibalism rates. For example, paired individuals of different instar stages of the antlion *Myrmeleon hyalinus* resulted in cannibalism in up to 75% of the cases^8^, and 20% of the diet of two *Pardosa* spider species consists in conspecifics^78^. This result fits well with our current finding of only a limited superiority of large individuals. One explanation for this could be the lack of obvious weaponry in wormlions, in contrast to the mandibles or chelicerae of antlions and spiders. Wormlions display an atypical predatory lifestyle compared to other fly larvae. Although predatory fly larvae in other families are known, such larvae prey on the soft larvae or eggs of other insects (or snails), and not on well-defended prey, such as ants, which are common prey of wormlions^79^. Indeed, antlions can subdue larger ants than can wormlions of the same size^54^, which may also explain why large wormlions “cope” less well with small conspecifics than other TB predators. This is perhaps the best wormlions can do given their morphology. Note that the propensity of cannibalism we report under laboratory settings is almost certainly exaggerated, owing to high density and lack of refuge. It is necessary to evaluate cannibalism rates in the field in order to determine how common it really is.

There were several clear differences between the two sets of treatments with shaded/lit vs. deep/shallow sand microhabitats. First, while density had a similar effect on the final location in all sand depth treatments, more wormlions remained in their initial placement location in the shade choice treatment than when placed under full shade or light conditions. Regarding the pit area, the final location in deep or shallow sand had a stronger effect than the treatment per se, while the shade/light treatments were more important for the pit area than the final location. This fits previous studies indicating that while both deep sand and shade are preferred, sand depth affects the pit area more strongly^80^. The reason is probably that while deep sand is preferred in order to construct larger pits, shade is preferred in order to avoid exposure to high temperatures and desiccation^58,60^. Second, large wormlions remained either under shade or in deep sand, while small ones relocated more frequently to lit or shallow sand areas. The advantage deep sand provides for large individuals is much greater than for small individuals^61^, as demonstrated in the interactive effect of wormlion size and sand depth on pit area (Fig. 3c). In contrast, shade should be similarly important for large and small wormlions alike. Third, when large individuals were placed before the smaller ones, the pits constructed in deep sand were larger (a significant sand depth × treatment interaction; Fig. 3d), suggesting once more that deep sand is more important for large wormlions than small ones.

In summary, we have demonstrated here evidence of competition in wormlions over favorable ambush sites. While large wormlions possess an advantage over small individuals, this advantage is weaker compared to that in other TB predators. Future studies should examine not only intraspecific competition but also competition with other insects, such as antlions, occupying ambush sites of a similar nature. Pit-building antlions and wormlions sometimes co-occur^53,54^. However, antlions are usually rare within wormlion clusters in the Mediterranean area (^57^; Scharf I, pers. obs.), and antlions are probably superior competitors in direct interactions with wormlions. An intriguing question thus arises as to what makes antlions unable to invade the wormlions' typical habitats? The answer could be related to an abiotic constraint, or perhaps to the ability of wormlions to settle for lower prey availability or prey of smaller size.

## Supplementary Information

Supplementary Information.
